# Is the manual following response an attempt to compensate for inferred self-motion?

**DOI:** 10.1007/s00221-019-05607-x

**Published:** 2019-07-24

**Authors:** Yajie Zhang, Eli Brenner, Jacques Duysens, Sabine Verschueren, Jeroen B. J. Smeets

**Affiliations:** 1 Department of Human Movement Sciences, Vrije Universiteit Amsterdam, Amsterdam Movement Sciences, Amsterdam, The Netherlands; 2 Department of Kinesiology, FaBer, KU Leuven, Leuven, Belgium; 3 Department of Rehabilitation Sciences, FaBer, KU Leuven, Leuven, Belgium

**Keywords:** Binding problem, Reaching, Standing, Postural control, Adjustment, Goal-directed movement

## Abstract

If the surrounding of a visual target unexpectedly starts to move during a fast goal-directed hand movement, the hand reflexively moves along with it. This is known as the ‘manual following response’. One explanation for this response is that it is a compensation for inferred self-motion in space. Previous studies have shown that background motion gives rise to both postural responses and deviations in goal-directed hand movements. To evaluate whether compensation for inferred self-motion is responsible for the manual responses we examined whether galvanic stimulation of the vestibular system would give rise to similar deviations in hand movements. Standing participants tried to quickly tap on targets that were presented on a horizontal screen. Participants could infer self-motion on some of the trials, either from galvanic vestibular stimulation or from background motion. Both perturbations took place during the hand movement. It took both the head and hand about 45 ms longer to respond to background motion than to respond to galvanic stimulation. The head responded in a similar manner to both types of perturbations. The hand responded about as expected to galvanic stimulation, but much more vigorously to background motion. Thus, the manual response to background motion is probably not a direct consequence of trying to compensate for inferred self-motion. Perhaps the manual following response is a consequence of an error in binding motion information to objects.

## Introduction

The manual following response is a reflexive response of the hand to sudden motion in the background when making a goal-directed movement. The hand is diverted in the direction of the background motion within about 150 ms after the background starts to move (Brenner and Smeets 1997; Whitney et al. 2003; Gomi et al. 2006). Manual following responses occur when people are sitting, as well as when they are standing on stable (Zhang et al. 2018b) or unstable (de Dieuleveult et al. 2018) surfaces. The manual following responses are present when moving to a memorised position (Whitney et al. 2003; Saijo et al. 2005) as well as when moving to a visible target that does not move with the background (Brenner and Smeets 1997; Zhang et al. 2018b).

It has been suggested that the manual following response arises from a mechanism that compensates for perceived self-motion (Gomi 2008). When standing, maintaining one’s balance is essential for interacting with the external world (Massion 1994). People combine visual, vestibular, and haptic information with knowledge about their voluntary movements to maintain their balance. Many of the mechanisms for maintaining balance are referred to as being reflexive because of their short latencies. Surface perturbations can elicit a myotatic stretch reflex after around 50 ms, which can be modified via a transcortical loop within 120 ms (Nashner 1976). Vestibular signals related to head motion can elicit muscle activity at latencies as short as 50 ms (Fitzpatrick et al. 1994; Forbes et al. 2016). That postural responses to a moving visual background (Saijo et al. 2005; Zhang et al. 2018b) are reflexive is evident from the fact that they have very short latencies and cannot be suppressed. Seeing the whole background move relative to oneself suggests that one oneself has moved, because that is more likely than that the whole world has moved.

The manual following response may be a consequence of reflexive postural compensations for self-motion: if the hand has to reach a stationary target, it should move in the opposite direction of any self-motion, just as the rest of the body will do (Gomi 2008). Therefore, irrespective of the basis on which self-motion is inferred, one expects to observe a response in the hand that resembles the postural response. The self-motion that can be inferred from lateral visual motion is a combination of rotation and translation, with the forehead moving in the opposite direction than the motion of the background. Therefore, effective manual and postural compensations should be in the same direction as the moving background, which is indeed the case (Zhang et al. 2018b).

There are two reasons to question the validity of the explanation of the manual following response that is given in the previous paragraph. The first is that visually induced postural responses are generally based on an analysis of motion in the whole visual field, whereas the manual following response is primarily based on background motion close to the target (Abekawa and Gomi 2010; Brenner and Smeets 2015). The second reason is that if the manual following response compensates for the same inferred self-motion as other postural responses, their amplitudes should be comparable. In our previous study the manual responses were much more prominent than the postural ones. We therefore decided to perform an explicit test of the inferred self-motion hypothesis.

For our explicit test of the inferred self-motion hypothesis we reasoned that the manual response to inferred self-motion should be independent of the sensory modality that is suggesting self-motion. If two stimulations induce similar postural responses, they are likely to have induced similar inferred self-motion, so they should also induce similar manual responses. We used galvanic vestibular stimulation (GVS) to suggest self-motion without moving the background or actually moving the participant.

The effects of GVS on apparent self-motion have been well documented (reviewed by Fitzpatrick and Day 2004; Cathers et al. 2005; Mian et al. 2010; St George and Fitzpatrick 2011; Kwan et al. 2019). Bipolar GVS gives rise to apparent self-motion (yaw and roll: forehead and top of the head towards cathode); and to apparent inter-aural linear acceleration (towards cathode). The apparent self-motion that this generates is the result of combining these distorted vestibular signals with unperturbed proprioceptive and visual signals. The apparent self-motion triggers a compensatory postural response (including a head response) to maintain balance (Britton et al. 1993; Fitzpatrick et al. 1994; Day et al. 1997).

As we are interested in the very first responses, and perception at very short timescale is very difficult to assess, we cannot directly equate or compare the inferred self-motion induced by our transient vestibular and visual perturbations. We will therefore use the postural response of the head to approximately match the magnitudes of inferred visual and vestibular self-motion. Thus, we will select magnitudes of GVS and background motion that induce a comparable pattern of head responses during goal-directed hand movements. If the manual following response is indeed a component of a general mechanism that compensates for self-motion, the manual following response should be similar for GVS as for background motion.

## Materials and methods

### Participants

Twenty young adults (29 ± 3 years, 12 males) participated in this study. Three of them self-reported being left-handed. All participants had normal or corrected-to-normal vision, and had no disease that is known to affect motor or sensory function. This study was approved by the research ethics committee at the Faculty of Behavioural and Movement Sciences, Vrije Universiteit Amsterdam. Written informed consent was obtained from each participant.

## Experimental setup

**Fig. 1:**
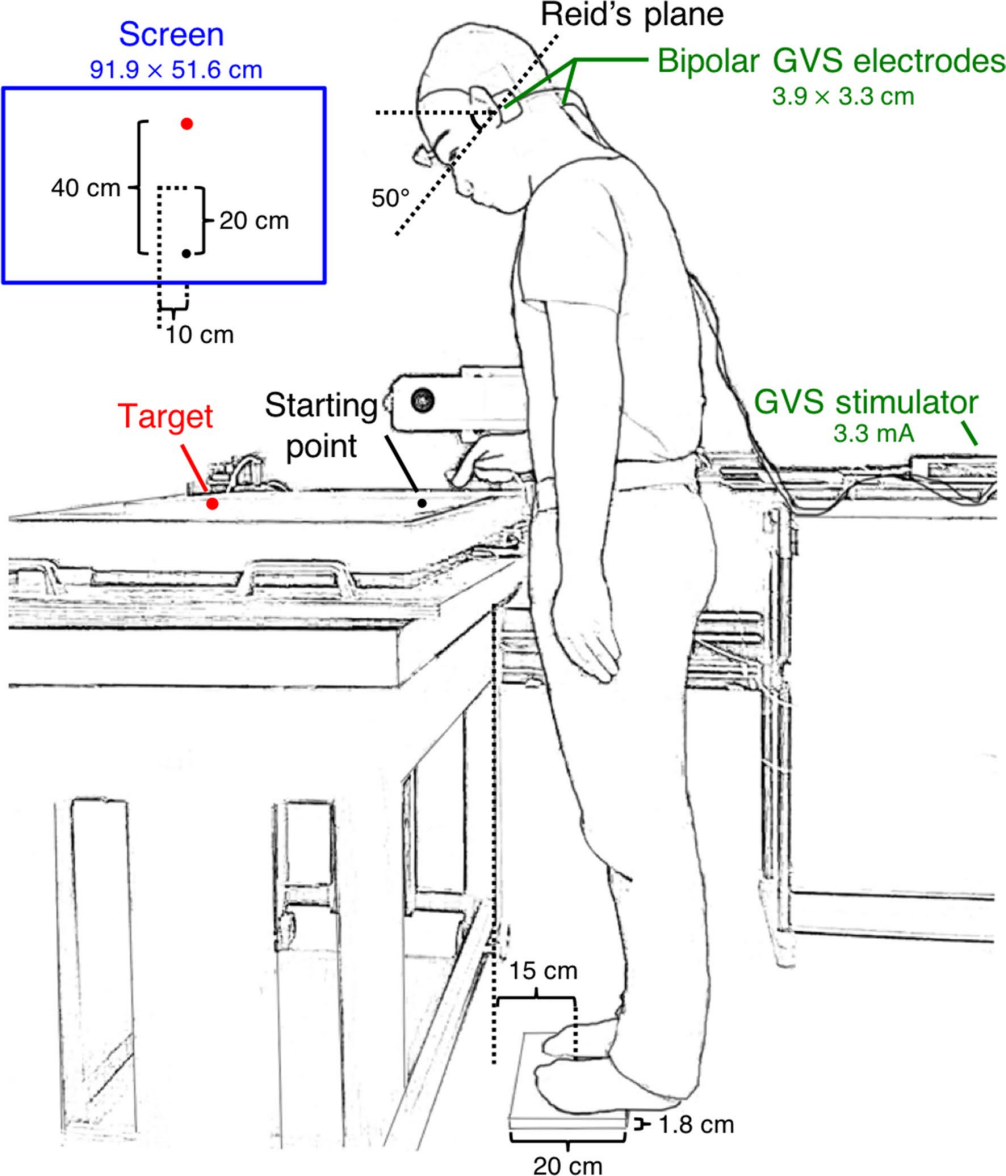
A participant standing in the setup before a trial starts. His head is pitched down. He stands with bare feet on a piece of wood. The visual stimulus is presented on a horizontal screen placed in front of him. The blue box shows a top-view of the screen

The setup is very similar to that used in previous research in our lab (Zhang et al. 2018b). Participants stood in front of a horizontal screen (60 Hz refresh rate, 91.9 × 51.6 cm, 1920 × 1080 pixel resolution) lying flat, face-up on a height-adjustable table (Fig. 1). Table height was adjusted to align the screen with the participant’s hip. The participants stood barefoot near the edge of the screen, with the front half of their feet on a piece of wood (height: 1.8 cm, width: 20 cm, centre to table edge: 15 cm) and the heels not touching the ground. This was done to challenge their balance. A starting point (radius: 1.5 cm, 20 cm closer than and 10 cm to the right of the screen centre) and a target (radius: 1.5 cm, 20 cm farther than and 10 cm to the right of the screen centre) were presented at different times on the screen during the task. When looking at the screen, the head pitched downwards by about 50°, as estimated from photographs of a side view of the starting posture. This head posture is known to enhance postural rotation around a vertical axis in response to GVS (Moreau-Debord et al. 2014). A cluster marker consisting of three markers attached rigidly to each other in a triangular configuration was attached to the forehead. This cluster was used to record the movements of the forehead. A rotation of the head around a vertical axis results in a lateral displacement of the forehead, and contributes thereby to our primary measure of head motion. We used the cluster to check the actual orientation of the head. The 95% confidence interval of the orientation of Reid’s plane with respect to the direction of gravity was 43°–57°.

A single marker was attached to the nail of the index finger of the right hand. This marker was used to record the movement of the hand and for the online control of the experiment. An Optotrak 3020 motion capture system (Northern Digital, Waterloo, Ontario, Canada) sampling at 200 Hz was used to measure movements of the finger and head. The camera was located to the right of the participant (visible behind the participant in the side view of Fig. 1).

GVS was applied to participants’ left and right mastoid processes using a linear isolated stimulator (Stmisola, Biopac Systems, Inc., Goleta CA, USA) via gel-coated carbon rubber electrodes (3.9 × 3.3 cm) in a binaural bipolar configuration.

A photodiode was attached to the far-right corner of the screen to help synchronize events on the screen with the recorded movements (to within 5 ms). The timing of the GVS was synchronized by measuring the signals driving the GVS with the analog input channel of the Optotrak system (ODAU).

### Procedure

The participant started each trial by placing the right index finger at a starting point. A target appeared at a random time between 0.6 and 1.2 s later. The participant was instructed to tap on the target as fast as possible with the tip of the right index finger. As soon as the participant moved 5 mm from the starting point a perturbation was triggered. To check for overall changes in responses as a result of the different self-motion perturbations, we also included perturbations of the target itself (1 cm displacement, either leftwards or rightwards). GVS occurred about 10 ms after the perturbation was triggered. It took about 50 ms longer for the visual perturbations (target jump or background motion) to take place due to the delay in rendering images on the screen. If the target was hit (i.e., if the tapping position of the finger was within the target), a sound indicated success. Otherwise, the target drifted away from where the finger touched the screen.

**Fig. 2:**
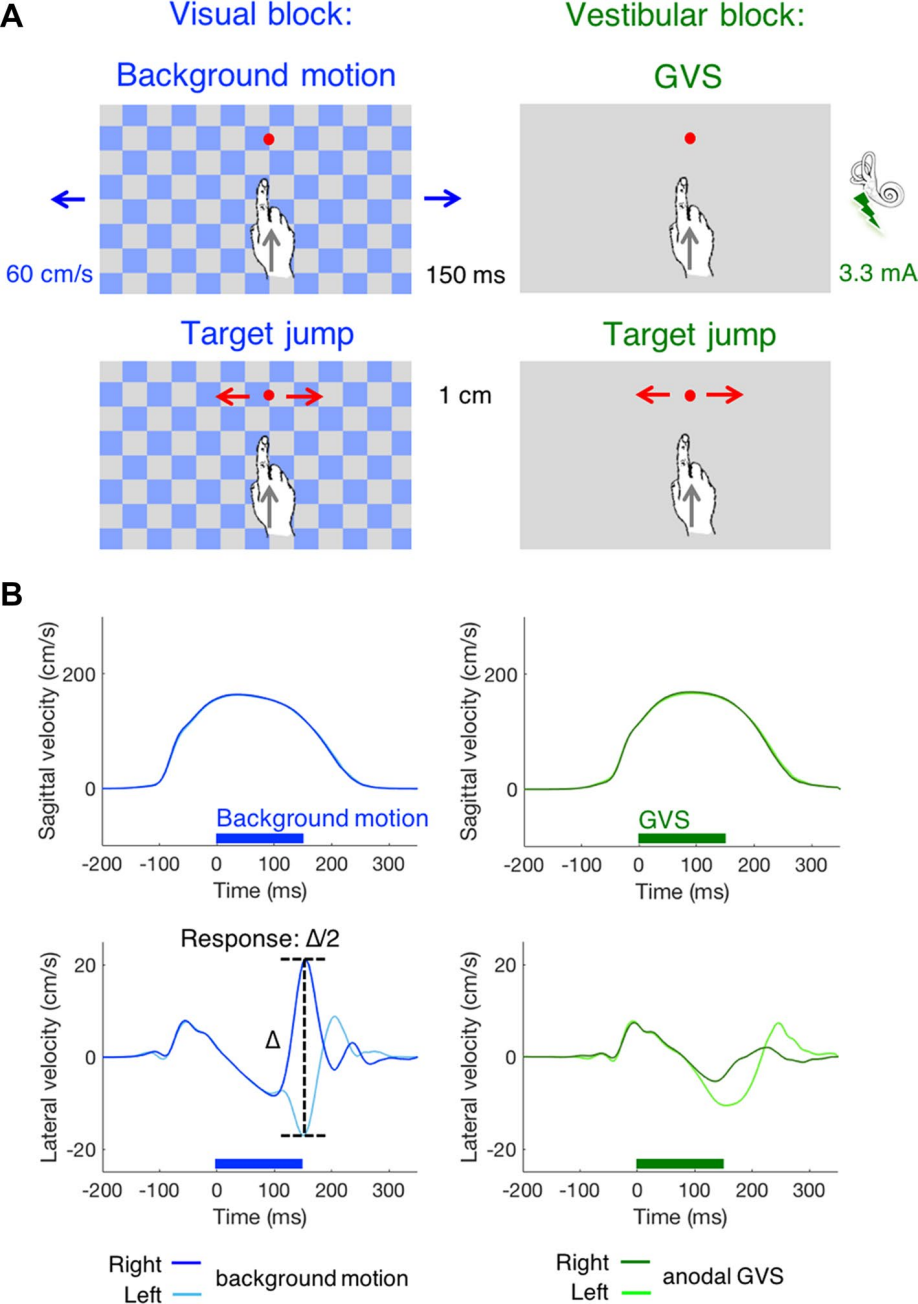
Conditions and a single participant’s average velocity profiles. **a** The two types of blocks (visual and vestibular) and two types of perturbation (induced self-motion and target jump). One of the two types of perturbation occurred on each trial within each block. Note that the size of the target jump (1 cm) is much smaller than the length of the red arrows. **b** The timing of the perturbation (horizontal bars) in relation to the average sagittal and lateral velocity profiles of a single participant

The experiment consisted of two blocks with background motion (visual blocks) and two blocks with galvanic stimulation (vestibular blocks; Fig. 2). A checkerboard-like background (square length: 7 cm) was continuously present during the visual blocks. In each trial of the visual blocks, either the background moved or the target jumped. When the background moved, it did so for 150 ms. It moved either leftwards or rightwards at 60 cm/s, ‘behind’ the stationary target. In the vestibular blocks, the background was uniformly grey to reduce possible visual suppression of the apparent self-motion. In each trial of the vestibular blocks either GVS was applied or the target jumped. The GVS consisted of a 3.3 mA DC current that was applied for 150 ms running from the left to right mastoid processes, or in the opposite direction.

The four blocks took place in a counter-balanced A–B–B–A design, which means that for 10 participants the first and fourth blocks were visual and the second and third blocks were vestibular, and the other 10 participants started and ended with a vestibular block. In case of any fatigue, between blocks and trials participants could rest at any time by not placing their finger on the starting point. The visual blocks had 100 trials of background motion (50 trials each with motion to the left and right). The vestibular block had 100 trials of GVS (50 trials each with the anode on the left and right). Both visual and vestibular blocks had 40 trials with target jumps (20 trials each with jumps to the left and right). The target jump trials served as a reference without illusory self-motion but with adjustments to the hand movement. The 140 trials of each block were presented in random order.

### Data analyses

The 3D kinematic data derived from the markers were filtered using a second-order low-pass Butterworth filter with a cutoff frequency of 30 Hz. We determined this cutoff frequency by determining the frequency at which the variance in the distances between the three markers on a cluster was minimal (Schreven et al. 2015). We excluded trials for which the trial duration in presenting the perturbation was not within ± 3 standard deviations of the mean. In total 1% of the trials were excluded.

Movement time was defined as the time from when the finger was lifted 5 mm above the screen until it tapped on the screen. Tapping error was defined as the lateral position of the tap with respect to the centre of the target. This error is signed: it is considered to be positive if it was to the side that we would expect given the direction of the perturbation (toward target jump, in the direction of background motion and towards the anode), and negative if it was to the other side. The main variable of interest is the response: how the lateral velocity profile was influenced by the perturbation. This variable is also defined as being positive when it is in the direction that we would expect for the perturbation. For this, lateral velocity profiles were determined for each direction of the perturbation, given the sign corresponding to that of the perturbation, and then averaged across the two directions. The response corresponds to half the difference between the two curves in the lower panels of Fig. 2b. We determined this response for both the hand and the forehead.

The latency of the average response to each kind of perturbation was determined with the extrapolation method (Veerman et al. 2008; Oostwoud Wijdenes et al. 2014). Following the methods used in our earlier studies (Zhang et al. 2018a, b), we defined the latency as the time at which a line through the points at 25% and 75% of the peak velocity of the response (when velocity is plotted as a function of time) crosses zero. The slope of this line was taken as a measure of the vigour of the response. Descriptive data are shown as mean values ± standard deviation across participants. Differences between the two types of blocks in the responses to target jumps were evaluated with a paired *t* test. In this test, *p* values smaller than 0.05 were considered to be significant.

## Results

Visual and vestibular blocks were each performed twice, in different orders. The tapping movements took 314 ± 43 ms in the visual blocks, and 285 ± 32 ms in the vestibular blocks. There was no systematic effect of the order of the blocks on the tapping errors or movement times (across participants). Neither were there systematic differences between the two repetitions of the same type of block (within participants). All the data for each kind of trial in each kind of block were therefore combined.

**Fig. 3:**
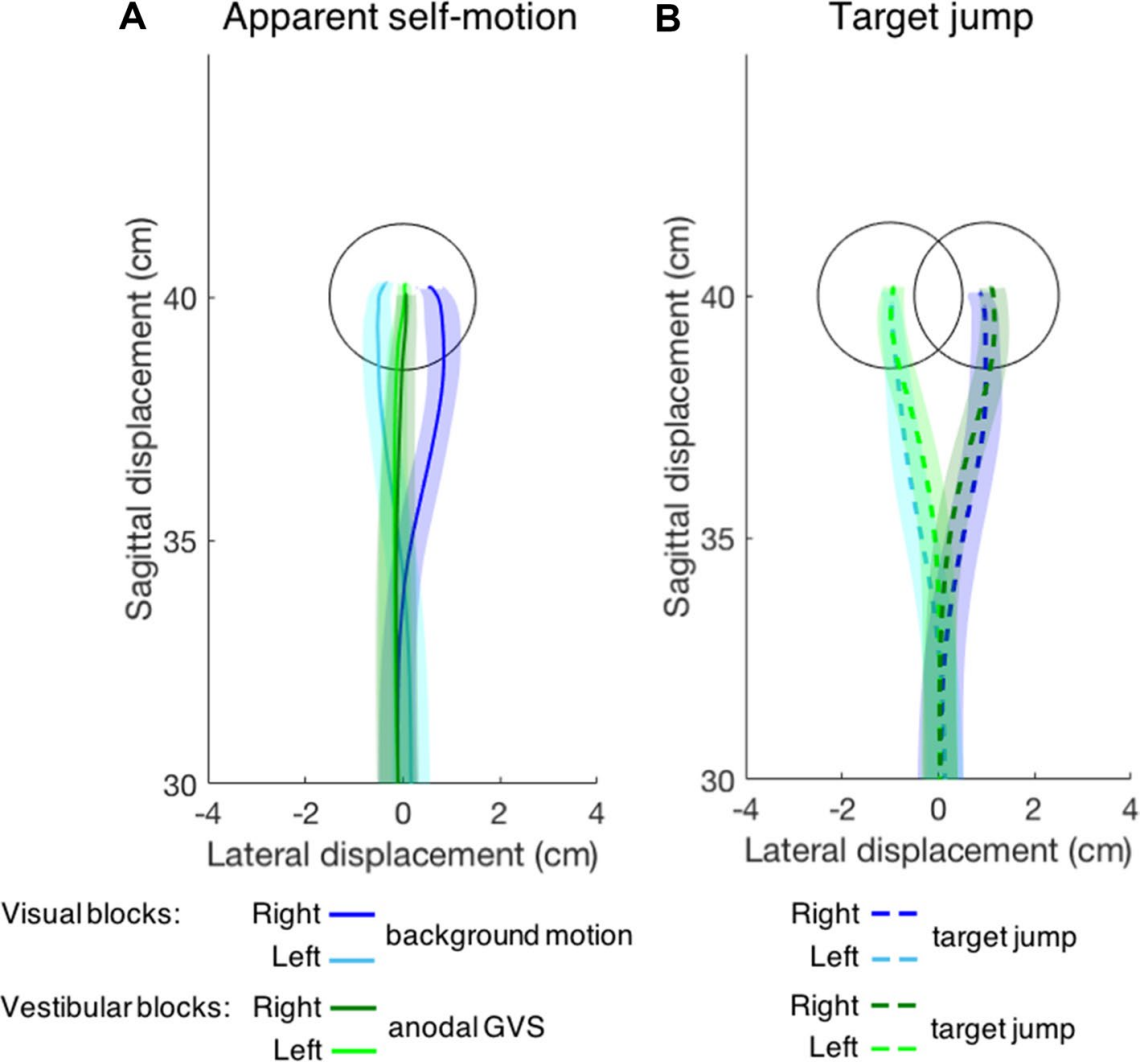
The final 10 cm of the mean hand trajectories in the horizontal plane when **a** the target remains stationary but perturbations induce apparent leftward or rightward self-motion, or **b** the target jumps. The hand started at (0, 0). Shaded areas represent the 95% confidence interval of the mean across participants

The average hand trajectories are affected differently by the two types of stimulation that were intended to cause equivalent apparent self-motion (Fig. 3a). The hand clearly followed the direction of background motion to the left or right (blue traces), while it was hardly influenced by the GVS (the two green traces are very close to each other). This smaller response of the hand to GVS than to background motion is not due to a general reduction of sensorimotor sensitivity in the vestibular blocks, because the hand moves correctly the new target location when the target jumps in both the visual and vestibular blocks (Fig. 3b).

In accordance with the hand’s trajectory only clearly being affected by the visually induced self-motion (Fig. 3a), participants made larger errors when confronted with background motion than when confronted with galvanic stimulation despite the longer movement times in the visual block. The tapping error was 0.47 ± 0.20 cm after background motion, while it was only 0.06 ± 0.05 cm after GVS. As expected based on the trajectories in Fig. 3b, the tapping errors after target jumps were small, and comparable in the visual and vestibular blocks (0.08 ± 0.20 cm and 0.10 ± 0.18 cm, respectively).

**Fig. 4:**
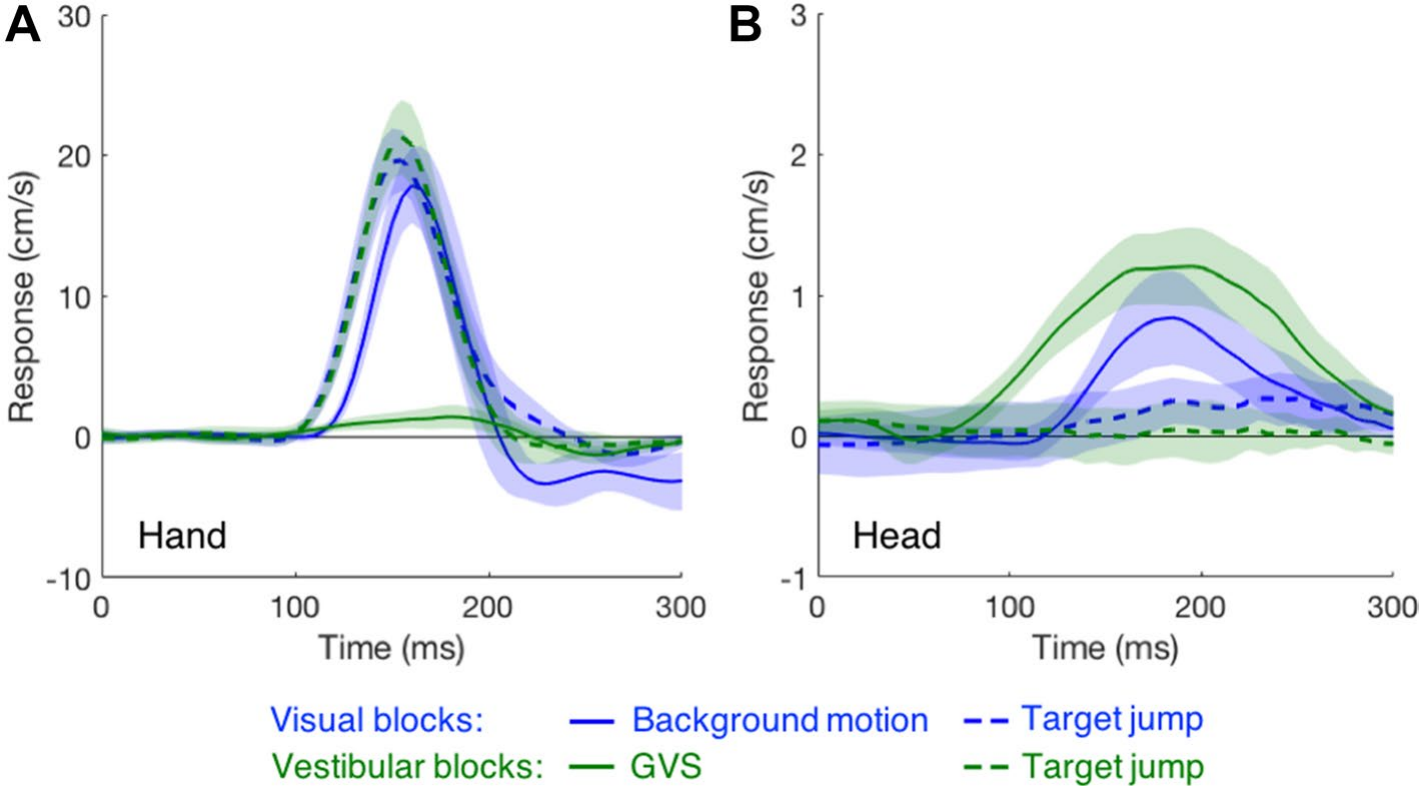
The signed effect of the perturbation on the lateral velocity profile of the **a** hand and **b** head as a function of the time after the perturbation. Shaded areas represent the 95% confidence interval across participants

The difference between the hands’ responses to background motion and to GVS can be observed in more detail when expressed as a function of time (Fig. 4a). The hand responded almost as vigorously to background motion as to a target jump, though with a slightly longer latency (124 ± 6 ms vs 112 ± 6 ms). The hand’s very small response to GVS started 77 ms after the perturbation and peaked at 1.5 cm/s (solid green trace in Fig. 4a). Neither the latency nor the vigour of the hands’ responses to target jumps differed significantly between the two types of blocks (dashed lines in Fig. 4a; visual: 112 ± 6 ms, 6.0 ± 1.5 m/s^2^; vestibular: 113 ± 7 ms, 6.5 ± 1.8 m/s^2^; *t*_19_ = 0.77, *p* = 0.45, *t*_19_ = 1.32, *p* = 0.20).

The head responded 119 ms after the onset of background motion, at about the same time as the hand did so (124 ms). The head’s response to the GVS started 81 ms after the onset of stimulation, again at about the same time as the hand did so (77 ms). Thus, it took both the head and the hand about 45 ms longer to respond to background motion than to respond to GVS, regardless of when the perturbation happened. The similar difference in delay for the head and the hand is in line with an explanation based on inferred self-motion. However, the much weaker response of the hand to GVS than to background motion cannot simply be due to the vestibular stimulation giving rise to less inferred self-motion, because the GVS induced a larger head response than did background motion (Fig. 4b). The head barely responded to the target jumps (Fig. 4b), while the hand responded more strongly to such jumps, indicating that the head’s response is indeed a response to inferred self-motion, or at least inferred motion of the head, rather than being a postural response that accompanies the hand’s response.

**Fig. 5:**
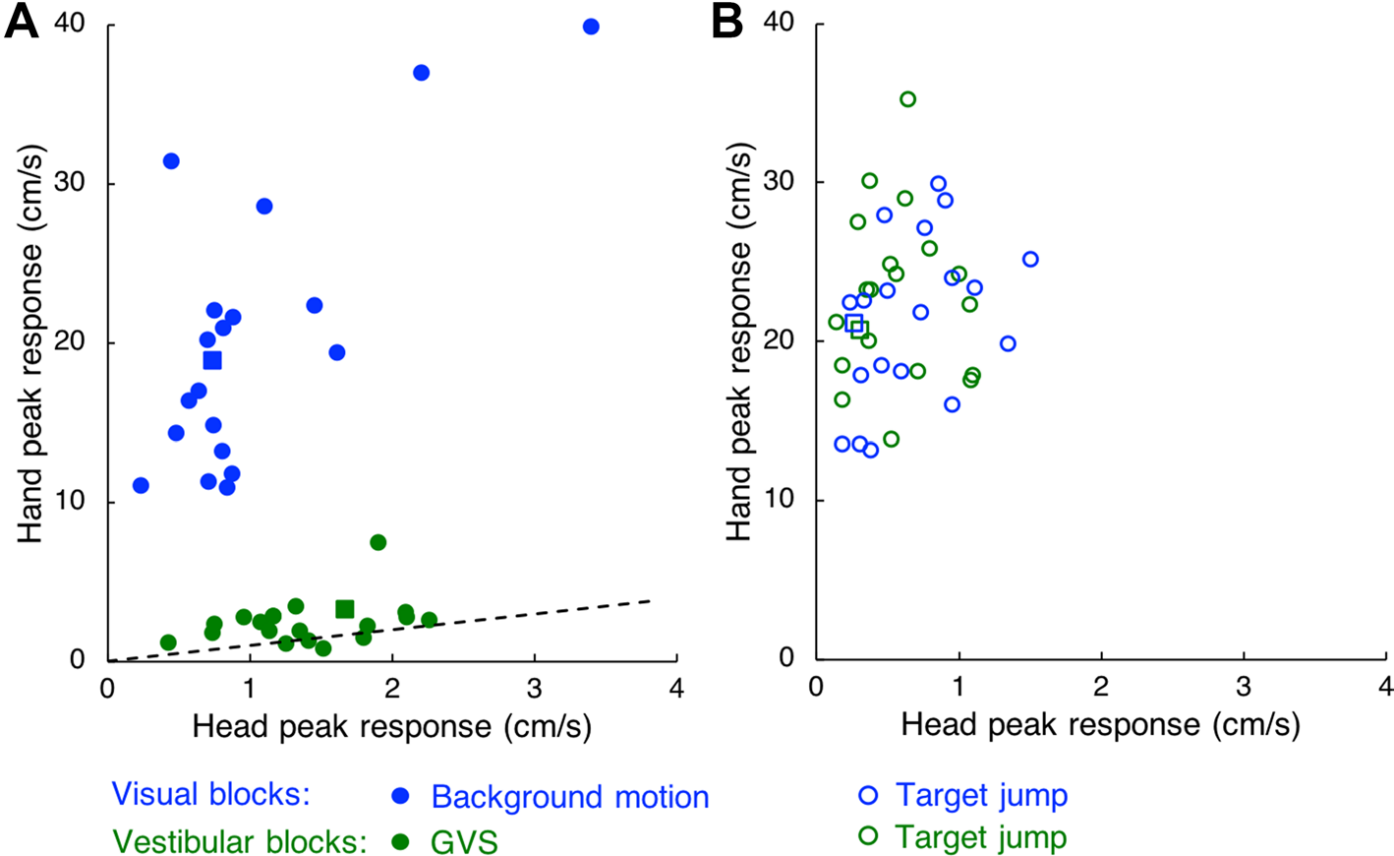
The peak value of each participant’s hand response as a function of his/her peak head response for the visual and vestibular blocks. The participant whose data are displayed in Fig. 2b is indicated by squares; the other participants by circles. **a** Responses to perturbations that induce apparent self-motion. The black dashed line indicates equal responses of head and hand, which corresponds to compensation for inferred self-motion for both visual and vestibular blocks. **b** Responses to target jumps

If the response of the hand is a compensation for inferred self-motion, we expected the hand to respond as much as the head to inferred lateral sway (dots at black dashed line in Fig. 5a). The predicted response of the hand will be larger than that of the head if the participants infer they are rotating around a yaw axis. The peak values of the responses of the participants’ hands were related to those of their heads both for background motion and for GVS (blue and green dots in Fig. 5a), but the responses were much stronger for the hand than for the head for background motion. Not surprisingly, for target jumps the peak responses of the participants’ hands were stronger than those of the heads, and were very similar in the visual and vestibular blocks (blue and green circles in Fig. 5b).

A much larger response of the hand than of the head would fit the self-motion hypothesis if GVS primarily gave rise to inferred lateral sway while background motion predominantly gave rise to inferred yaw rotation. We therefore determined the rotational responses to both kinds of perturbations, and found that the peak yaw response was on average about 5°/s. Such rotation would add a few cm/s to the predicted hand’s velocity, possibly explaining why the slope for the GVS response in Fig. 5 was above 1.0. As the maximal effect on arm velocity (arm fully stretched at peak response) would be 5 cm/s, this effect cannot explain the hand responses to background motion.

## Discussion

We examined whether compensation for inferred self-motion could explain the manual following response to background motion. The self-motion that participants inferred from our very short visual and vestibular perturbations could not be assessed directly. We assumed that the head response to both perturbations aimed to compensate for the inferred self-motion. As these responses were comparable for the two perturbations (Fig. 4b), the manual responses should also have been comparable if they were based on the inferred self-motion. However, the manual responses were very different (Fig. 4a): the hand response to the vestibular perturbation was appropriate to counteract the inferred self-motion, but the response to background motion was an order of magnitude too strong (Fig. 5a). This suggests that the manual following response is not simply a compensation for inferred self-motion. The difference in the hand response is due to the difference in stimulation, rather than other differences between the blocks (e.g., the grey rather than checker-board background, or a 20 ms longer movement time) because the hand response to target jumps was similar for vestibular and visual blocks (dashed green and blue traces in Fig. 4a).

The hand response to GVS was small compared to that to background motion (Fig. 4a), but its absolute value (1.5 cm/s) was comparable to that in other studies (Bresciani et al. 2002; Moreau-Debord et al. 2014; Keyser et al. 2017). However, our responses to GVS have a much shorter latency: 77 ms rather than more than 200 ms after GVS onset in the other studies. A notable difference between our study and theirs is that our participants were standing with the head free, whereas the participants’ heads were fixed in the other studies: either sitting with the head supported (Moreau-Debord et al. 2014; Keyser et al. 2017) or standing with a bite-board (Bresciani et al. 2002). It is well known that such restraints have a profound influence on sensorimotor responses (Steinman et al. 1990). Furthermore, our participants had continuous vision of the target, whereas reaches were made without any vision of target and hand in the other studies. It is known that the gain of fast responses can be modified (Day and Lyon 2000; Smeets et al. 2016). Fixing the head prevents self-motion, and thus removes the need for online postural control. In a similar fashion, removing the target makes it unnecessary to strive for a high gain for the online control. Consequently, the participants in studies with head fixed and target removed had difficulties in reaching the target accurately, whereas our participants did not. We also used a shorter stimulation duration (150 ms rather than about 400 or 500 ms), but this cannot explain the difference as the remaining movement time after the stimulus stopped was only about 120 ms.

As we anticipated longer latency responses to GVS than to background motion (based on the literature discussed in the previous paragraph), we did not delay the GVS stimulation to correct for the slow response of the display, so stimulation was earlier for GVS than for background motion. The latency of online adjustment is independent of the moment of perturbation, but the intensity of the response increases as the remaining time until the end of the movement decreases (Oostwoud Wijdenes et al. 2011; Zhang et al. 2018a). This could have given rise to a slightly stronger response of the hand to self-motion induced by background motion than by vestibular stimulation. Such a difference would be reduced by the longer movement times in the visual block, but might be increased by the longer latency to respond to background motion. In any case, it is unlikely that adjustments to the remaining time could account for the order of magnitude stronger response of the hand to background motion than to galvanic stimulation (Fig. 4a).

In contrast with the expected longer latency for the manual response to GVS than to background motion, the response latencies of both the hand and the head to GVS were about 45 ms shorter than those to background motion. This is (in hindsight) logical because vestibular neuronal delays are shorter than visual delays, partly due to the temporal response properties of the retina (Scheich and Korn 1971). The short latency of the hand’s responses to GVS rules out the possibility that the responses are caused by retinal slip as a result of vestibulo-ocular reflexes (Schneider et al. 2002) or as a result of the head responses (Migliaccio et al. 2013). The hand’s response to GVS might be not specific to the goal-directed movement, but part of a postural adjustment that aims at restoring balance after an inferred perturbation. This adjustment ensures that gaze and hand remain directed towards the target.

We reject the inferred self-motion hypothesis on the basis of the too large responses of the hand to background motion. What could be the basis of the manual following response? Any direct coupling between hand and head can be rejected, because when the target jumped the head moved much less for comparable hand movements (Fig. 5b).

An alternative explanation for the manual following response is based on findings in studies that varied the part of the background that moved. In those studies, it was found that the manual responses are most sensitive to visual motion near the target or near where the target had been (Abekawa and Gomi 2010; Brenner and Smeets 2015). How can we explain these results? A possible direction might be to regard the manual following response as the consequence of a ‘binding’ error. The underlying idea is that distributing the coding of different properties across separate areas of the visual cortex gives rise to the need to bind such properties when they are relevant for the task at hand (Treisman 1996). For the experiments on background motion, many motion-sensitive receptive fields that include the region occupied by the target are likely to be larger than the target. The consequence is that they will sense motion of the background as well. Therefore, the brain might detect sudden motion at the location of the target, attribute this motion to the target, and follow the motion vigorously with the hand. The motion may only be correctly linked to the background after receiving feedback from higher visual areas that separate the target from its background (Lamme and Roelfsema 2000). In accordance with this possibility, similar manual responses have been found when obstacles near the target move (Aivar et al. 2008).

In conclusion, we directly compared the responses of the hand and head to self-motion inferred from visual and vestibular stimulation. We found that the hand does not compensate even nearly as vigorously to vestibular as to visual stimulation when the stimulation is chosen so that the head responds similarly. Therefore, the manual response to background motion cannot just be a compensation for perceived self-motion.

## Supplementary material

Raw data used in this study are available on Open Science Framework at https://osf.io/nzu6h/ (10.17605/osf.io/nzu6h).

## References

[CR1] Abekawa N, Gomi H (2010). Spatial coincidence of intentional actions modulates an implicit visuomotor control. J Neurophysiol.

[CR2] Aivar MP, Brenner E, Smeets JBJ (2008). Avoiding moving obstacles. Exp Brain Res.

[CR3] Brenner E, Smeets JBJ (1997). Fast responses of the human hand to changes in target position. J Mot Behav.

[CR4] Brenner E, Smeets JBJ (2015). How moving backgrounds influence interception. PLoS One.

[CR5] Bresciani JP, Blouin J, Popov K, Bourdin C, Sarlegna F, Vercher JL, Gauthier GM (2002). Galvanic vestibular stimulation in humans produces online arm movement deviations when reaching towards memorized visual targets. Neurosci Lett.

[CR6] Britton TC, Day BL, Brown P, Rothwell JC, Thompson PD, Marsden CD (1993). Postural electromyographic responses in the arm and leg following galvanic vestibular stimulation in man. Exp Brain Res.

[CR7] Cathers I, Day BL, Fitzpatrick RC (2005). Otolith and canal reflexes in human standing. J Physiol.

[CR8] Day BL, Lyon IN (2000). Voluntary modification of automatic arm movements evoked by motion of a visual target. Exp Brain Res.

[CR9] Day BL, Cauquil AS, Bartolomei L, Pastor MA, Lyon IN (1997). Human body-segment tilts induced by galvanic stimulation: a vestibularly driven balance protection mechanism. J Physiol.

[CR10] de Dieuleveult AL, Brouwer AM, Siemonsma PC, van Erp JBF, Brenner E (2018). Aging and sensitivity to illusory target motion with or without secondary tasks. Multisens Res.

[CR11] Fitzpatrick RC, Day BL (2004). Probing the human vestibular system with galvanic stimulation. J Appl Physiol.

[CR12] Fitzpatrick R, Burke D, Gandevia SC (1994). Task-dependent reflex responses and movement illusions evoked by galvanic vestibular stimulation in standing humans. J Physiol.

[CR13] Forbes PA, Luu BL, Van der Loos HF, Croft EA, Inglis JT, Blouin JS (2016). Transformation of vestibular signals for the control of standing in humans. J Neurosci.

[CR14] Gomi H (2008). Implicit online corrections of reaching movements. Curr Opin Neurobiol.

[CR15] Gomi H, Abekawa N, Nishida S (2006). Spatiotemporal tuning of rapid interactions between visual-motion analysis and reaching movement. J Neurosci.

[CR16] Keyser J, Medendorp WP, Selen LPJ (2017). Task-dependent vestibular feedback responses in reaching. J Neurophysiol.

[CR17] Kwan A, Forbes PA, Mitchell DE, Blouin JS, Cullen KE (2019). Neural substrates, dynamics and thresholds of galvanic vestibular stimulation in the behaving primate. Nat Commun.

[CR18] Lamme VA, Roelfsema PR (2000). The distinct modes of vision offered by feedforward and recurrent processing. Trends Neurosci.

[CR19] Massion J (1994). Postural control system. Curr Opin Neurobiol.

[CR20] Mian OS, Dakin CJ, Blouin JS, Fitzpatrick RC, Day BL (2010). Lack of otolith involvement in balance responses evoked by mastoid electrical stimulation. J Physiol.

[CR37] Migliaccio AA, Della Santina CC, Carey JP (2013). Transmastoid galvanic stimulation does not affect the vergence-mediated gain increase of the human angular vestibulo-ocular reflex. Exp Brain Res.

[CR21] Moreau-Debord I, Martin CZ, Landry M, Green AM (2014). Evidence for a reference frame transformation of vestibular signal contributions to voluntary reaching. J Neurophysiol.

[CR22] Nashner LM (1976). Adapting reflexes controlling the human posture. Exp Brain Res.

[CR23] Oostwoud Wijdenes L, Brenner E, Smeets JBJ (2011). Fast and fine-tuned corrections when the target of a hand movement is displaced. Exp Brain Res.

[CR24] Oostwoud Wijdenes L, Brenner E, Smeets JBJ (2014). Analysis of methods to determine the latency of online movement adjustments. Behav Res Methods.

[CR25] Saijo N, Murakami I, Nishida S, Gomi H (2005). Large-field visual motion directly induces an involuntary rapid manual following response. J Neurosci.

[CR26] Scheich H, Korn A (1971). Timing properties and temporal summation in retina. Pflugers Arch Eur J Physiol.

[CR27] Schneider E, Glasauer S, Dieterich M (2002). Comparison of human ocular torsion patterns during natural and galvanic vestibular stimulation. J Neurophysiol.

[CR28] Schreven S, Beek PJ, Smeets JBJ (2015). Optimising filtering parameters for a 3D motion analysis system. J Electromyogr Kinesiol.

[CR29] Smeets JBJ, Oostwoud Wijdenes L, Brenner E (2016). Movement adjustments have short latencies because there is no need to detect anything. Mot Control.

[CR30] St George RJ, Fitzpatrick RC (2011). The sense of self-motion, orientation and balance explored by vestibular stimulation. J Physiol.

[CR31] Steinman RM, Kowler E, Collewijn H (1990). New directions for oculomotor research. Vis Res.

[CR32] Treisman A (1996). The binding problem. Curr Opin Neurobiol.

[CR33] Veerman MM, Brenner E, Smeets JBJ (2008). The latency for correcting a movement depends on the visual attribute that defines the target. Exp Brain Res.

[CR34] Whitney D, Westwood DA, Goodale MA (2003). The influence of visual motion on fast reaching movements to a stationary object. Nature.

[CR35] Zhang Y, Brenner E, Duysens J, Verschueren S, Smeets JBJ (2018). Effects of aging on postural responses to visual perturbations during fast pointing. Front Aging Neurosci.

[CR36] Zhang Y, Brenner E, Duysens J, Verschueren S, Smeets JBJ (2018). Postural responses to target jumps and background motion in a fast pointing task. Exp Brain Res.

